# Phytochemical Constituents, Antioxidant, Cytotoxicity, Antimicrobial, Antitrypanosomal, and Antimalarial Potentials of the Crude Extracts of *Callistemon citrinus*

**DOI:** 10.1155/2019/5410923

**Published:** 2019-08-28

**Authors:** Rotimi Larayetan, Zacchaeus S. Ololade, Oluranti O. Ogunmola, Ayodele Ladokun

**Affiliations:** ^1^Department of Pure and Applied Chemistry, University of Fort Hare, Alice 5700, South Africa; ^2^Department of Chemistry, Kogi State University, Anyigba, Nigeria; ^3^Department of Chemistry, University of Medical Sciences, Ondo, Nigeria; ^4^Department of Chemistry, Emmanuel Alayande College of Education, Oyo State, Nigeria; ^5^Department of Geography and Environmental Science, University of Fort Hare, Alice 5700, South Africa

## Abstract

Plants are reservoir for potentially useful bioactive compounds, and owing to the rising occurrences of drug resistance to malaria parasites, there is a need to discover and develop new phytochemicals in plant that can be used as antimalarial agents. In this study, we gave a detailed description of the phytochemicals present in both ethyl acetate and methanolic extracts of *Callistemon citrinus* (*C. citrinus*) using Gas Chromatography-Mass Spectrometry (GC-MS) analysis; both extracts were also evaluated for their *in vitro* antimalarial, antitrypanosomal, and cytotoxicity activities against *Trypanosoma brucei brucei* (*T. b brucei*) parasites, *Plasmodium falciparum* (*P. falciparum*) malaria parasites 3D7 strain, and human cervix adenocarcinoma cells (HeLa cells); in addition, the antimicrobial and antioxidant efficacies were determined using standard methods. Both extracts were characterized by a high amount of fatty acids (52.88 and 62.48%). The ethyl acetate extract exhibited a greater activity with minimum inhibitory concentration (MIC) values ranging from 0.025 to 0.10 mg/mL while the methanol extract ranged from 0.025 to 0.15 mg/mL. Both extracts were bactericidal to *Escherichia coli ATCC 35150* (*E. coli*) and *Pseudomonas aeruginosa ACC* (*P. aeruginosa*). Qualitative and quantitative phytochemical screenings conducted for both extracts revealed the presence of alkaloids, glycosides, saponins, steroids, and triterpenoids, fat and oils, flavonoids, phenols, and tannins in varying amounts. Both crude extracts exhibited antitrypanosomal potentials with an IC_50_ of 6.6/9.7 *μ*g/mL and antiplasmodial activities with an IC_50_ of 8.4/13.0 *μ*g/mL. Conclusion from this study indicates that apart from the folkloric uses of this plant in traditional settings, the extracts possess a broad spectrum of antimicrobial, antitrypanosomal, and antimalarial activities and some pharmaceutically essential bioactive components with remarkable antioxidant capacities that may be used in the synthesis of novel drugs for the management of different varieties of ailments.

## 1. Introduction

Plants are used by humans to ease and treat several diseases. Nowadays, in several countries of the world, traditional medicines are used as a substitute to conventional medicine [1, 2]. Countless medicinal plants possessing antioxidant activity now draw the attention of many researchers to investigate their role in the fight against numerous diseases like Alzheimer's disease, cancer, atherosclerosis, cerebral cardiovascular events, diabetes, and hypertension to mention just a few [3, 4]. Plants serve as reservoir for potentially valuable chemical compounds which can be used to produce drugs resulting into lead molecules used for current design and synthesis [5, 6]. Plant extracts together with their phytochemicals possess antimicrobial properties which are of great importance for therapeutic treatment [7]. Most pharmacological activities of medicinal plants are traced to their secondary metabolites which are smaller molecules when compared to the constituents of primary metabolites like proteins, carbohydrates, and lipids. Secondary metabolites like alkaloids, terpenoids, tannins, saponins, flavonoids, and cardiac glycosides from both medicinal and aromatic plants can be used for the synthesis of various antimicrobial and antifungal drugs which are relatively less harmful to man [8]. Plants with medicinal basis are usually utilized by traditional practitioners in most rural areas of developing countries of the world [9, 10]. *C. citrinus* is an aromatic and medicinal plant of the family Myrtaceae and a shrub endemic to Australia. It is a potential medicinal plant used in local settings for the treatment of gastrointestinal distress, pain, and infectious diseases from bacteria, fungi, viruses, and parasites [11]. It is also used as an herbicide [12] while its leaves are used as a tea substitute with a revitalizing pleasant flavour [13]. Extracts from this plant are also used to treat bronchitis and respiratory conditions like cough. It is used as an insecticide, and its essential oil is used as an antimicrobial and antifungal agent [14, 15]. Records about the fungitoxicity and antinociceptive activity of the leaves of this plant have been documented [16, 17]. It has been established that both human and animal trypanosomiasis adversely affect the sum total economy of Africa via the deteriorating health recorded for human and animals [18]; in addition to this, the unavailability of vaccines that can be used against trypanosomiasis and the increasing cost of commencing and sustaining tsetse fly control have resulted to most infected areas of Africa to be dependent on the use of trypanocidal drugs [19] which has also exhibited some resistance [20] coupled with record of toxicity, inefficiency, and exorbitant price [21]. *Plasmodium falciparum*, the parasite that causes malaria, a disease that creates a higher threat in several parts of the world, have been documented to show resistance to nearly all classes of antimalarial drugs in clinical use [22]. Consequently, there is the need for a safer, efficient, and inexpensive therapeutics that can be obtained from the bioactive compounds from plant. Phytochemical analysis carried out on the leaves of this plant revealed the presence of alkaloids, terpenoids, steroids, and flavonoids [23]. Cock [24, 25] documented the antimicrobial properties of *C. citrinus* against different pathogens of Gram-positive, Gram-negative, and fungi strains. Several researchers from across the globe have reported the essential oil components of this plant [16, 26–30]. On the contrary, there is a drought of information as regards the comparative assessment of the antioxidant, antibacterial, antimalarial, and antitrypanosomal activities and particularly the bioactive components of the crude extracts of *C. citrinus* which necessitated this present study.

## 2. Materials and Methods

### 2.1. Plant Material

Fresh leaves of this plant were collected from their natural habitat in the premises of the University of Fort Hare (UFH), Eastern Cape, South Africa, in August 2018. This was authenticated by a taxonomist in Botany Department of the university, and a voucher sample (Larayetan 1) was kept in the Giffen Herbarium of University of Fort Hare for record purpose.

### 2.2. Microbial Strains

Two resistant reference strains of bacteria and six resistant bacteria were obtained from the laboratory stock culture of AEMREG in Biochemistry and Microbiology Department of the university. All the cultures were maintained on nutrient agar until further use. The reference and laboratory strains are three Gram-positive bacteria *Staphylococcus enteritis (ACC)*, *Staphylococcus aureus (ACC)*, and *Listeria monocytogenes (ACC)* and five Gram-negative bacteria *Aeromonas hydrophila (ACC)*, *Escherichia coli (ATCC 35150)*, *Salmonella typhi (ACC)*, *Vibrio alginolyticus (DSM 2171)*, and *Pseudomonas aeruginosa ACC*.

### 2.3. Analytical Reagents Used

Reagents and chemical used in this research include the following.

2,2-Azinobis-(3-ethylbenzothiazolin-6-sulfonic acid) diammonium, 2,2-diphenyl-1-picrylhydrazyl (DPPH), and potassium persulfate (PPS) sourced from Sigma-Aldrich (St Louis, USA); Mueller-Hinton agar from Oxford Ltd. (Hampshire, England); ethyl acetate, methanol, and dimethyl sulfoxide (DMSO) were purchased from Fluka Chemicals (Buchs, Switzerland). All chemicals and reagents employed were of analytical grade.

### 2.4. Preparation of Plant Extracts

Fresh leaves of the plant were air-dried for 21 days at room temperature. The dried leaves were pulverized by using polymix (PX-MFC-90D), a mechanical grinder; the resulting powder was used to prepare two different extracts as highlighted in the following.

#### 2.4.1. Methanol Extract

Two hundred and fifty grams of the powdered dried leaves was soaked in 800 mL of methanol for 72 hours; the mixture was shaken on an orbital shaker (Model 420 Series, Thermo Fisher Scientific) at 250 rpm, filtered with Whatman No. 1 (320 mm, 4 *μ*m) filtered paper, and concentrated at low pressure using a rotary vacuum evaporator (bath at 40°C). The concentrate was preserved in a vial, labelled appropriately, and stored in the refrigerator at 4°C until needed for analysis.

#### 2.4.2. Ethyl Acetate Extract

Two hundred and fifty grams of the powdered dried leaves was equally soaked in 800 mL of ethyl acetate for 72 hours. The same procedure as described in Section 2.4.1 was followed to obtain dry ethyl acetate extract, which was also stored in a vial and kept in the refrigerator until further analysis.

### 2.5. GC-MS Determination of Bioactive Compounds

Presence of bioactive components in the two extracts was revealed by GC-MS examination carried out by multidimensional gas chromatography coupled with a mass spectrophotometer (Shimadzu Japan, 2010) as previously described [31, 32]. This machine possesses polar doubled capillary and nonpolar column (25.0 m × 0.25 *μ*m i.d., 0.25 *μ*m df). Helium of high purity was used as the carrier gas at a flow rate of 0.99 mL/minutes. Starting and final temperatures were 60°C and 280°C at a heated rate of 3°C/minute which was constant isothermally for 6 minutes; solvent cut time was set at 3 minutes; EI mode was 70 eV while linear velocity for the column was set at 36.8 cm/second. Recognition of the bioactive components in both the ethyl acetate and methanol extracts of the plant under study was done by harmonizing their mass spectra data (MSD) with those obtained from both NIST library mass spectra and the literature; furthermore, the retention index (RI) of the individual constituent was correlated with those obtained in the literature.

### 2.6. Qualitative Phytochemical Screening

The ethyl acetate and methanol extracts of the plant were subjected to examination for the detection of phytochemical compounds by employing the procedures of Harborne [33] and Evans [34]. The qualitative detection of the various phytochemicals was carried out by using Mayer's and Wagner's reagents (alkaloids). Other tests carried out include the modified Keller–Killiani test for glycosides, foam test (saponins), Salkowski and Liebermann Burchard's tests (steroids and triterpenoids), stain test (fat and oil), and ferric chloride (phenols and tannins) and lead acetate (flavonoids) tests.

#### 2.6.1. Quantification of Total Tannin Content

Tannin determination was evaluated according to the method depicted by Van Buren and Robinson [35] with slight alteration as illustrated by Kaur and Arora [36] using tannic acid as standard. Two hundred and fifty milligrams (250 mg) of the extracts was added to 50 mL of distilled water in a conical flask. The mixture was agitated for 1 h by using a mechanical shaker and subsequently filtered into a 50 mL volumetric flask and made up to the final volume by addition of distilled water. An aliquot (1 mL) of the filtrate was mixed with 4 mL of distilled water and treated with 2 mL (10-fold dilution) of 0.1 M FeCl_3_ in 0.1 M HCl and 0.008 M potassium ferrocyanide. The resultant solution was mixed thoroughly and allowed to stay for 10 minutes; the absorbance was measured at 605 nm against the blank. The quantification was carried out based on the 7-point standard calibration curve of tannic acid (20, 40, 60, 80, 100, 140, 200 mg/L) in distilled water. The tannin content was articulated as tannic acid equivalents (TAEs) in milligram per 100 grams of the dry material.

#### 2.6.2. Phosphomolybdate Assay

The total antioxidant ability of *C. citrinus* extracts was examined by phosphomolybdate technique with ascorbic acid as standard [37]. A portion (0.1 mL) of the sample extracts was added with 1 mL of reagent solution containing 0.6 M sulphuric acid, 28 mM sodium phosphate, and 4 mM ammonium molybdate. Different tubes containing the mixture were covered with an aluminium foil and incubated in a water bath at 95°C for 90 min. The test tubes were brought out of the water bath and allowed to cool to ambient temperature; the absorbance of the mixture was then measured at 765 nm against the blank, using ascorbic acid as the standard.

#### 2.6.3. Quantification of Total Phenolic Content

The phenolic contents of both extracts were examined via a spectrophotometric method [38]. About 1 mg/mL of each extract in 1 mL of solvent was added to 1 mL of Folin–Ciocalteu reagent in different test tubes, and the mixture was left for about 4 minutes, and then, 10 mL of 7% Na_2_CO_3_ solution and 13 mL of deionized distilled water were added to the above mixture. The tubes were vortexed for about 25 seconds and kept in the dark at 25°C for colour development; absorbance was read at 750 nm. The analyses were done in triplicate, and the results were expressed as mg GAE/100 g of gallic acid using a prepared calibration curve with linear equation presented as follows: *y* = 0.009*x* + 0.012 (*R*^2^ = 0.999), where *x* is the concentration and *y* is the gallic acid equivalent.

#### 2.6.4. Quantification of Total Flavonoid Content

The method of Ordonez [39] was employed to determine the total flavonoid content. About 0.5 mL of 2% AlCl_3_ in ethanol solution was mixed with 0.5 mL of the extracts and kept at 25°C for 1 h; absorbance was measured at 420 nm, and the flavonoid content was expressed as mg RE/100 g of rutin using the equation as follows: *y* = 0.023*x* + 0.022 (*R*^2^ = 0.982), where *x* is the concentration and *y* is the rutin equivalent.

#### 2.6.5. Quantification of Total Flavonol Content

The evaluation of the total flavonol content in the leaf extracts was done in accordance with the method of Kumaran [40], in which 2.0 mL of the sample, 3.0 mL of sodium acetate (50 g/L), and 2.0 mL of aluminium trichloride prepared in ethanol were mixed together. The absorbance of the mixture was measured at 440 nm after 2.5 h at 20°C. Total flavonol content was then estimated as mg QE/100 g of quercetin equivalent (QE) from the calibration curve using the equation: *y* = 0.003*x* − 0.003, *R*^2^ = 0.998, where *x* is the concentration and *y* is the quercetin equivalent.

### 2.7. Antibacterial Assay

#### 2.7.1. Minimum Inhibitory and Bactericidal Concentrations (MIC and MBC)

The minimum inhibitory concentration (MIC) of methanol and ethyl acetate extracts were determined through the microdilution procedure illustrated by Collin et al. [41] To achieve this, 750, 800, 850, 900, and 950 *μ*L of Mueller-Hinton Broth (MHB) was distributed to each one of the eppendorf tube and stock solutions of both methanol and ethyl acetate extracts (20 mg/mL) were prepared in DMSO. Aliquots of 250, 200, 150, 100, and 50 *μ*L of both extracts were dispensed into each tube having the MHB to raise the final volume to 1000 *μ*L, respectively. Precisely, 25 *μ*L of the inoculums' suspension from each bacterial strain (0.5 McFarland, ∼1 × 108 cfu/mL) was subsequently mixed and vortexed to permit for adequate mixing of both extract and broth. Each eppendorf tube was labelled appropriately and incubated for a day at 37°C. The experiment was carried out in duplicate. Ciprofloxacin and DMSO were employed as both positive and negative control. The MIC of the extracts was described as the smallest concentration that depicts no observable growth when put side by side with the control containing only MHB, while the minimum bactericidal concentration (MBC) was determined by the pour plate method of all tube content devoid of any noticeable growth in the MIC method above onto fresh Mueller-Hinton agar plates and the culture was then incubated for 24 h at 37°C. The smallest concentration of extracts that did not reveal any colony growth on the exterior of the solid medium after an incubation period of 24 h was considered as the MBC.

### 2.8. *In Vitro* Antioxidant Action

#### 2.8.1. DPPH Assay

The radical-scavenging and antioxidant activities of ethyl acetate and methanol crude extracts were estimated alongside the free radical DPPH.

Various concentrations ranging between 0.025 and 0.40 mg/mL of the extracts and commercial antioxidant (vitamin C) were incubated with a DMSO solution of DPPH for about 30 minutes at room temperature in the dark. A vortex machine was employed to give a thorough vibration of the mixture, and the absorbance was read at 517 nm. The capacity of the crude extracts to hunt for DPPH free radical was calculated using the equation as follows: % inhibition = (A_control_ − A_vo_)/(A_control_) × 100, where A_control_ is the absorbance of DPPH + DMSO and A_vo_ is the absorbance of DPPH + crude extracts or the commercial antioxidant [25]. The dose-response curve was plotted, and the IC_50_ value of the commercial antioxidant and crude extracts was calculated [42].

#### 2.8.2. ABTS Assay

The modified technique of Nantitanon et al. [43] was employed to assess the ABTS potencies of both ethyl acetate and methanol extracts.

The operational solution was acquired by oxidation of ABTS stock solution (7 mM) with 2.4 mM of potassium persulfate in equal amounts, and the blend was allowed to react for 12 h at room temperature. To a fraction of the ensuing solution (1 mL), 60 mL of methanol was added to it and absorbance reading at 734 nm (0.706 ± 0.001) after 7 minutes was taken via a UV-spectrophotometer. Briefly, the various concentrations ranging from 0.025 to 0.4 mg/mL) of each of the extract were added to the methanol solution of ABTS for 7 minutes at ambient temperature in the dark. Absorbance was afterward estimated spectrophotometrically at 760 nm, and the ABTS % inhibition through the extracts and commercial antioxidant (vitamin C) was calculated by means of the equation portrayed for DPPH assay above.

### 2.9. Cytotoxicity Activity

The cytotoxicity of methanolic and ethyl acetate of *C. citrinus* crude extracts was examined by means of human cervix adenocarcinoma cells (HeLa cells) as depicted by Keusch et al. [44]. The stock solution of these two extracts (20 mg/mL) were dissolved in dimethyl sulfoxide (DMSO) and diluted with a culture medium. The resulting mixtures above were incubated in duplicate wells with about 1 × 10^4^ HeLa cells per well for 48 h at 37°C in 5% CO_2_. The amounts of cells which survive contact with the drugs were estimated through resazurin-based reagent and evaluating resorufin fluorescence in a multiwell-plate reader. The obtained results were expressed as percentage viability.

### 2.10. Antimalarial Activity

The antiplasmodial assay was carried out through parasite lactate dehydrogenase (*pLDH*) on malaria parasite *P. falciparium* as described by Makler et al [45]. Chloroquine obtained from Sigma-Aldrich was employed as positive controls at a concentration of 20 *μ*M. The stock solutions for the screening of methanol and ethyl acetate crude extracts against malaria parasites were carried out the same way as described in the cytotoxicity assay. 50 *μ*g/mL of the crude extracts was added with parasite culture in 96-well plates; these were incubated for 48 h at 37°C in a 5% CO_2_ incubator. At the end of the 48 h, the pLDH assay was carried out by removing 20 *μ*L of the culture from each well and added to 125 *μ*L of a combination of Malstat and nitrotetrazolium blue chloride (NBT)/phenazineethosulphate (PES) solutions in a fresh 96-well plate. The purple colour produced when pLDH is present is determined by absorbance at 620 nm which is equivalent to the number of parasites in the well. The extract that is able to decrease parasite viability appreciably from the single concentration assay is further employed in a dose-response assay to determine IC_50_ values.

### 2.11. Antitrypanosomal Activity

To access antitrypanosomal activity, stock solutions were also prepared the same way as depicted in the cytotoxicity assay. Fifty *μ*g/mL of the two extracts was added to the *in vitro* culture of *Trypanosoma brucei brucei* (*T. b brucei*) in 96-well plates, and the resultant mixtures were incubated for 48 h; the number of parasites that are capable to withstand the drug contact was calculated by adding resazurin-based reagent as explained earlier in cytotoxicity assay. The reagent resazurin is usually reduced to resorufin by living cells. Pentamidine was used as a positive control [46].

### 2.12. Statistical Analysis

Statistical analysis data was analysed using Microsoft Excel and reported as the mean ± standard deviation of triplicate determinations. In addition to this, nonlinear regression using Prism 5 for Windows, Version 5.02 (Graph Pad Software, Inc) program, was used to resolve IC_50_ from the dose-response curve [46].

## 3. Results and Discussion

### 3.1. Constituents of the Extracts

Twenty and twenty-two bioactive compounds representing 98.73 and 99.98% were found in the methanol and ethyl acetate extracts of the plant after GC-MS examination, and their percentage yield were 4.36 and 8.95%, respectively. The retention indexes, molecular formula, molecular weight, peak area (%), and nature of the compounds are presented in Tables 1 and 2. Although, the level of fatty acids found in the two extracts was high (52.88 and 62.48%); it is apparent that the main compounds characterizing both extracts are qualitatively and quantitatively different. Other components of the methanol and ethyl acetate extracts include esters (21.48 and 8.06%), oxygenated monoterpenoids (8.00 and 4.48%), and triterpenes (2.98 and 2.52%), respectively. Oleic acid, an omega-9-fatty acid, was one of the major components from the GC-MS results of the two extracts (6.42 and 28.23%). It is found in various animal and vegetable sources; it is a medium for drugs and other active ingredients in the pharmaceutical industry and acts as an emulsifying agent in aerosol product. The dietary importance of oleic acid in a balanced diet has been largely discussed by researchers, with a serious emphasis on the cardiovascular system. Oleic acid has been found to be essential for the brain [47] with several health benefits; it is used as an additional constituent in the preparation of cosmetics [48]. It is capable of thwarting ulcerative colitis [49], defending cells from free radical damage [50], reducing blood stress [51], and enhancing fat burning [52]. Palmitic acid, a saturated long-chain fatty acid comprising sixteen carbon atoms, was also found abundantly in both methanol and ethyl acetate extracts of our plant of study (32.87 and 13.57%). It is one of the major and mostly spread normal saturated acids found in plants like palm oil, palm kernel oil, odoriferous plants, *Moringa oleifera* seed oil, in animals and animal-derived products like cheese, milk, and meat, and microorganisms [52]. It is also used in the production of cosmetics [53]. Stearic acid, a saturated fatty acid having 18-carbon chain, was present in a substantial amount in both methanolic and ethyl acetate extracts of the plant (13.59 and 10.43%). It is also called octadecanoic acid and is mostly employed in the manufacturing of detergent, soaps, and cosmetics like shampoos and shaving cream products. Soap is not obtained directly from stearic acid but through the saponification of triglycerides containing stearic acid esters. Surfactants, cosmetics, and personal hygiene products are obtained from stearic acid [54]. Stearic and palmitic acids when taken in moderation exhibit anti-atherosclerotic and mild antioxidant activities in animal models [55]. It is noteworthy to say that squalene, a triterpenoids compound synthesized in the human liver, was also obtained in both extracts at varying amounts. The antioxidant and chemopreventive activity of squalene against colon carcinogenesis has been documented [56, 57]. It was found in a relatively lower amount in both extracts of *C. citrinus* (2.98 and 2.52%). The various components of both extracts of the plant of study have been linked to different medicinal actions. Eucalyptol, an oxygenated monoterpenoid, was found in an appreciable amount in both the methanol and ethyl acetate extracts of this study (2.22 and 4.55%); this component is known for its bronchodilator, anti-inflammatory, and mucolytic and mucociliary potentials as documented in our previous study [25]. It has been reported to have encouraging effects on the lung function parameters whether for the common cold or continual obstructive pulmonary ailment [58]. It also has bacteriostatic and bactericidal potencies [59]. These set of bioactive compounds found in both extracts of this plant possess synergistic effects which may be accountable for the therapeutic benefits of *C*. *citrinus* as employed by traditionalists.

### 3.2. Phytochemical Screening

The phytochemical study of the ethyl acetate and methanol plant extracts both revealed the presence of different bioactive compounds such as alkaloids, saponins, steroids, and triterpenoids, fats and oils, flavonoids, phenols, and tannins (Table 3). Bioactive compounds stored in plant possess biological and antibacterial activities that can be used as an alternative medicine for the treatment of bacterial infections in man [60]. These compounds have been reported to bestow resistance in opposition to microbial pathogens and this could be accountable for the exhibition of antibacterial activity by both extracts in this present study [61]. Also, secondary metabolites like terpenoids have been reported to have anti-inflammatory, antimalarial, antibacterial, and antiviral activities and reported to inhibit cholesterol synthesis [62]. Alkaloids are believed to have a broad range of pharmacological potentials like antimalarial, antiasthma, and anticancer properties [63]. Saponins act as an expectorant and are used in the management of upper respiratory tract inflammations; they furthermore possess antidiabetic and antifungal properties [64]. Previous phytochemical studies on the leaf of *C. citrinus* revealed the presence of alkaloids, flavonoids, terpenoids, and steroids [23].

#### 3.2.1. Total Tannin Content (TTC)

Tannins are polyphenolic compounds found to be present in different plant parts [65]. It has been reported that tannin displays antioxidant, antimicrobial, and anti-inflammatory properties [66]. Eating food rich in tannin can offer a lot of curative and beneficial effects to man. The TTC in the ethyl acetate extract was 12,000 ± 65.34 mg TAE/100 g while the level of tannin in the methanol extract was 8,000 ± 28.67 mg TAE/100 g. Overall tannin content was higher in the ethyl acetate extract than methanol extract as shown in Table 4.

#### 3.2.2. Total Antioxidant Capacity (TAC)

The total antioxidant capacity is a quantitative means to determine the degree of reduction of Mo(VI) to Mo(V). The TAC of both ethyl acetate and methanol samples in this study is 1568.73 ± 61.03 and 3031.53 ± 133.07 mg AA/100 g, respectively (Table 4). The antioxidant ability of ascorbic acid was employed as a reference standard with which plant extracts with potential antioxidant were compared [67].

#### 3.2.3. Total Phenolic Content (TPC)

Phenolic substances are ubiquitous secondary metabolites in plants. They are known to possess antioxidant activity [68–70]. The result obtained from this study shows that the leaf extracts of *C. citrinus* contained phenolic compounds and the content was higher in the methanolic extract than the ethyl acetate extract. This might be due to the influence of extraction solvent on the overall content of the phenolic compounds. The TPC in both ethyl acetate and methanolic leaves extract of this study were found to be 10,964.11 ± 40.22 and 22,511.23 ± 105.12 mg GAE/100 g, respectively (Table 4). The results were higher than those documented for the same species from India (261 mg/g) [71], possibly because of the influence of environmental factors on the phenolic content.

#### 3.2.4. Total Flavonoid Content (TFC)

Flavonoids, a secondary metabolite that refers to a class of naturally occurring polyphenols, are found in plants. They are utilized in the manufacture of pigments that attract insects for pollination in plants. They cannot be synthesized by animals and man because they are phytochemicals [72]. They are usually accountable for taste, colour, impediment of fat oxidation and prevention of enzymes, and vitamins degradation in food [73]. In addition to all these, they also exhibit significant anti-inflammatory, anti-allergic, and anti-cancer activities [74]. The most abundant flavonoids in food are the flavonols. The flavonoid content of the crude extracts was determined with reference to the standard rutin and expressed as its equivalent (mg·RE/g). The TFC obtained in both ethyl acetate and methanol leaf extracts was 368.12 ± 14.48 and 688.37 ± 39.92 mg·RE/100 g, making polar methanol extract of this present study higher than ethyl acetate as shown in Table 4.

#### 3.2.5. Total Flavonol Content (TFlC)

Flavonols are light yellow and weakly soluble substances found in leaves, fruits, berries, and flowers of 80% higher plants. The evaluation of TFlC in the extracts of the plant of study was expressed as quercetin equivalent. The highest flavonol content as presented in Table 4 was observed in the methanol extract (512.90 ± 11.00 mg·QE/100 g), while the lowest content was seen in the ethyl acetate extract (438.38 ± 11.73 mg·QE/100 g).

### 3.3. Antioxidant Activities of the Crude Extracts

**Figure 1:**
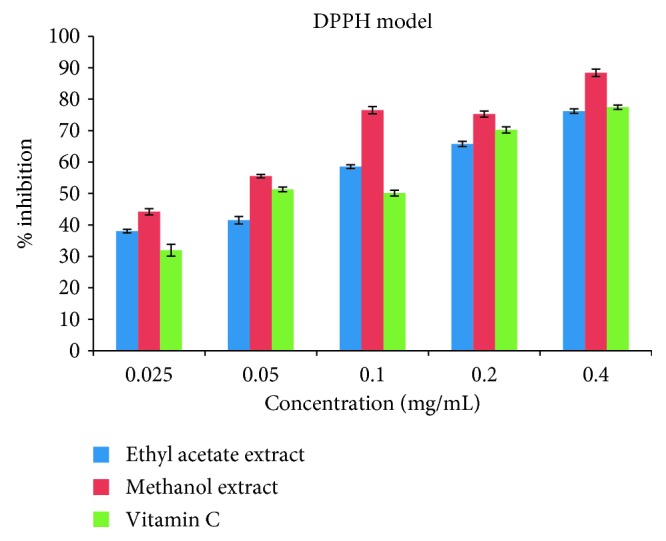
Antiradical effects of ethyl acetate and methanol extracts of *Callistemon citrinus* and standard drug (vitamin C) on DPPH radicals.

**Figure 2:**
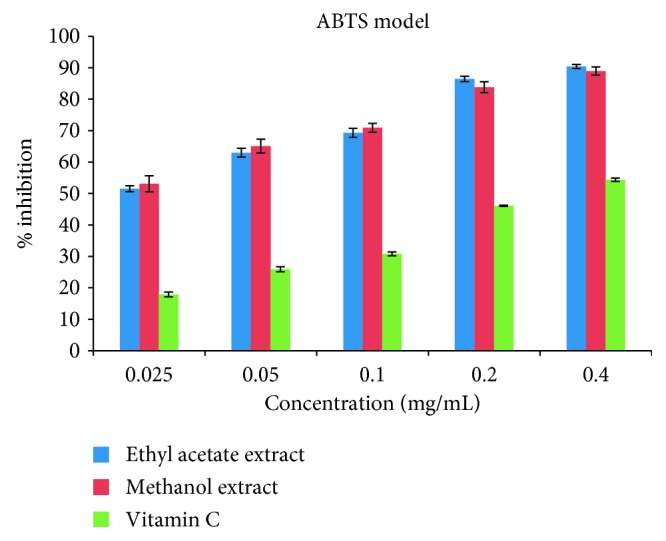
Antiradical effects of ethyl acetate and methanol extracts of *Callistemon citrinus* and standard drug (vitamin C) on ABTS radicals.

The DPPH• antioxidant assay is based on the principle that any substance capable of donating an atom of hydrogen or an electron is an antioxidant or antiradical species and its potency is demonstrated as DPPH•. Colour is transformed from purple to yellow in the test sample owing to the formation of neutral DPPH-H molecule upon the uptake of a hydrogen atom from antioxidant species [75]. It has been documented that it is preferable to use up to two methods when carrying out a test on antioxidant activity [76]. The evaluation of the antioxidant activity of the ethyl acetate and methanol crude extracts was carried out *in vitro* through two radical models (DPPH and ABTS), and the antioxidant capacity of the two extracts was measured based on their efficient IC_50_ concentration which corresponds to the concentration of the extracts capable of reducing the initial DPPH• absorbance by 50%. The IC_50_ of the ethyl acetate extract (2.41 ± 0.25 mg/mL) was lower than that of the methanol extract (1.33 ± 0.24 mg/mL) in the DPPH assay, but the two extracts showed better activity than the standard drug (vitamin C) with an IC_50_ of 2.43 ± 0.49 mg/mL. In the case of the ABTS assay, the ethyl acetate extract with an IC_50_ of 0.80 ± 0.36 mg/mL was also found to scavenge the radicals less than the methanol extract having an IC_50_ of 0.52 ± 0.52 mg/mL. Like the DPPH assay, the two extracts under ABTS also exhibited a good activity than the standard drug (vitamin C) with IC_50_ (4.60 ± 0.24 mg/mL). A better and more efficient result was recorded for the ABTS assay as shown in the IC_50_ results for the two experiments (Table 4). Percentage inhibitions of these radicals by the extracts and reference standard (vitamin C) were concentration-dependent (0.025 to 0.4 mg/mL) articulated in the percentage inhibition versus concentration as illustrated in Figures 1 and 2.

### 3.4. Minimum Inhibitory Concentration (MIC) and Minimum Bactericidal Concentration (MBC)

The MICs and MBCs of the two extracts of *C. citrinus* against the microorganism tested are shown in Tables 5 and 6. The MIC is also helpful in ascertaining the level of resistance of a particular bacterial strain and thus serves as a pointer to the use of certain antimicrobial agents. Both methanol and ethyl acetate extract showed significant inhibitory activities against the two multidrug-resistant reference strains of *Escherichia coli (ATCC 35150)* and *Vibro alginolyticus (DSM 2171)* in addition to the six multidrug-resistant bacteria *Aeromonas hydrophila ACC*, *Salmonella typhi (ACC)*, *Pseudomonas aeruginosa (ACC)*, *Staphylococcal enteritis (ACC)*, *Staphylococcus aureus (ACC)*, and *Listeria monocytogenes (ACC)*. The ethyl acetate extract MIC values of 0.025 ± 0.00, 0.025 ± 0.01, 0.025 ± 0.01, and 0.025 ± 0.00 mg/mL showed more potent activities than the methanol extract with MIC values of 0.15 ± 0.01, 0.100 ± 0.00, 0.100 ± 0.00, and 0.100 ± 0.01 mg/mL against *Aeromonas hydrophila (ACC)*, *Vibro alginolyticus (DSM 2171)*, *Staphylococcal enteritis (ACC)*,and *Listeria monocytogenes (ACC)*. Both extracts of *C. citrinus* under this present study were bacteriostatic against *Aeromonas hydrophila (ACC)*, *Vibro alginolyticus (DSM 2171)*, and *Salmonella typhi (ACC)* as shown in Table 3, but it is interesting to see that ethyl acetate extract was bacteriostatic against *Staphylococcal enteritis (ACC)* at a concentration of 0.100 ± 0.00 mg/mL but its methanol counterpart was bactericidal at a lower concentration of 0.025 ± 0.01 mg/mL. The bactericidal activities of both ethyl acetate and methanol extracts on both Gram-negative *Pseudomonas aeruginosa (ACC)*, a multidrug-resistant bacterium at a concentration of (0.100 ± 0.00) mg/mL, and *Escherichia coli (ATCC 35150)*, multidrug-resistant reference strains also at the same concentration of (0.100 ± 0.00) mg/mL of the plant under investigation, further establish the findings obtained in our previous study [25] that volatile oil from the leaves and flowers of *C. citrinus* is more effective against Gram-negative bacteria. The high fatty acid content as shown in Tables 1 and 2 of both methanol and ethyl acetate extracts might also contribute to the excellent antimicrobial activity recorded in this work because previous documentation by these researchers [77–79] have attested to the fact that high fatty acid components might be responsible for the antibacterial, anti-inflammatory, and antiviral potentials of *Pentanisia prunelloides* and *Helichrysum pedunculatum*. In addition to this, the disparities seen in the antimicrobial properties of both ethyl acetate and methanol extracts might be due to discrepancy in the chemical constituents of the two extracts coupled with some bioactive compounds such as alkaloids, tannins, terpenoids, ether, and phenolic compounds like flavonoids, which are considered to be bacteriostatic and bactericidal as reported in our previous study [25]. *Callistemon citrinus* plant is used in folk medicine to treat gastrointestinal distress, bronchitis, respiratory conditions like cough and as an anti-inflammatory agent. The two extracts from this study demonstrated the strongest inhibitory properties against *P. aeruginosa* and *E. coli* as rightly stated above. *P. aeruginosa*, a recognised pathogen, is linked to chronic obstructive pulmonary disease associated with intense inflammation [80, 81], and *E. coli*, a member of Enterobacteriaceae family, is responsible for diseases like gastrointestinal and urinary tract infections [80–82]. The extracts also showed a good inhibitory effect for a Gram-positive bacterium (*S. aureus*) which is responsible for lower respiratory tract infection, ventilator-assisted pneumonia, and osteomyelitis [83]. Findings from this study point out that the leaves of *C. citrinus* contain antibacterial components that justify the traditional and medicinal usage of the plant to guard against infections caused by both Gram-positive and Gram-negative bacteria.

### 3.5. Antitrypanosomal Activity

**Figure 3:**
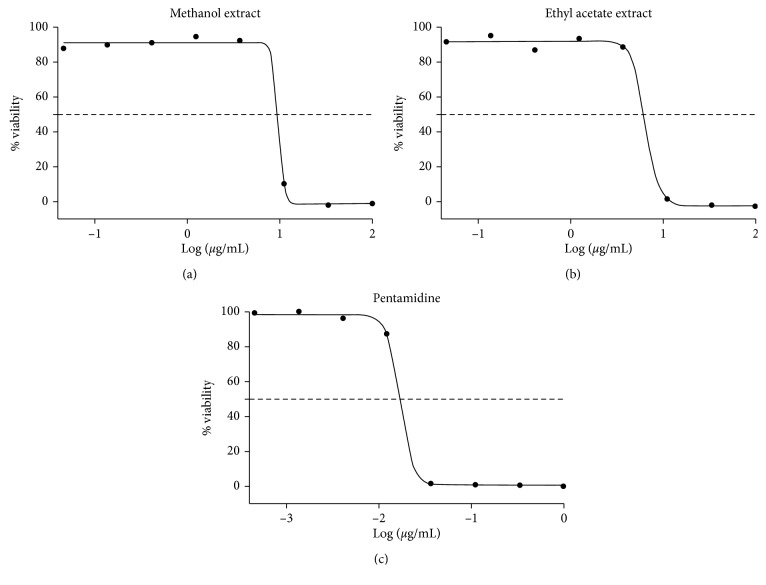
Dose-response curve for trypanosome assay of methanol and ethyl acetate extracts.

The methanol and ethyl acetate extracts of *C. citrinus* exhibited antitrypanosomal activity with IC_50_ values of 6.6 and 9.7 *μ*g/mL at a concentration of 50 *μ*g/mL as obtained from the dose-response curve shown in Figure 3. Both extracts showed better activities than the aqueous seed extract of our previous study that exhibited antitrypanosomal potential with an IC_50_ of 11.06 *μ*g/mL [84, 85] documented that the IC_50_ value of ≤20 *μ*g/mL is considered as excellent or very strong, whereas IC_50_ between 20 and 60 *μ*g/mL is regarded as moderate, but IC_50_ >100 *μ*g/mL is termed not active. This present study did not embark on the isolation of the different compounds that may be accountable for the observed antitrypanosomal activity, but the phytochemicals screening of both extracts showed that alkaloids, saponins, steroids, and triterpenoids, fat and oils, flavonoids, phenols, and tannins were present in the extracts and this might be attributed to the antitrypanosomal activity recorded for these two extracts [86]. In our previous study, the aqueous leaf, flower, and seed extract of *C. citrinus* showed antitrypanosomal activity with IC_50_ values of 11.06, 33.66, and 31.31 *μ*g/mL, respectively [87].

### 3.6. Antimalarial Activity

**Figure 4:**
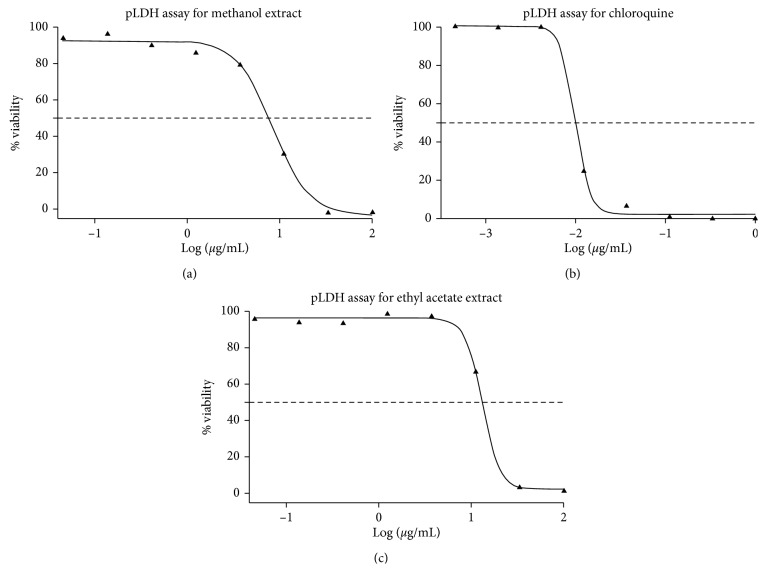
Dose-response curve for pLDH assay of methanol, ethyl acetate extracts, and chloroquine standard drug.

The methanol extract of *C. citrinus* at a concentration of 50 mg/mL greatly reduced the viability of *P. falciparium* to 0.00% with an IC_50_ of 8.4 *μ*g/mL (Figure 4). Furthermore, at the same concentration, the ethyl acetate extract appreciably reduced *P. falciparium* parasite to 35.72% with an IC_50_ of 13 *μ*g/mL (Figure 4). Samples which cause a significant decrease in PLDH to at least 50% were put forward for *pLDH* IC_50_ screening. Chloroquine that was employed as the reference drug exhibited an IC_50_ of 0.010 *μ*M. The crude aqueous extracts of the leaf, flower, and seed of *C. citrinus* plant from our previous study were tested against *P. falciparum* strain 3D7 parasite and found to be inactive as they were not able to decrease the % viability of the *P. falciparum* parasite [46]. There is a slight disparity in the antiplasmodial activity of the hydrophilic extract of this present study compared to those reported by Jenett-Siems et al. [87], who establish that lipophilic extracts were more active than hydrophilic extracts. Aqueous extracts usually prepared by the traditional practitioner are closer in composition to hydrophilic extracts of this study.

## 4. Conclusion

The inhibitory role observed on the various microorganisms and parasites by the crude extracts of *C. citrinus* coupled with the low cytotoxicity is an indication that it contains a broad spectrum of antimalarial, antimicrobial, and antitrypanosomal potentials and can be considered a prospective source of new drugs for the treatment of malaria and tropical ailment caused by microorganisms. This study has also provided rationale for the use of this plant not only in traditional medicine but also as a backup of scientific information to justify its different folkloric uses in traditional rural settings. Further studies are ongoing on this plant to isolate, categorize, characterize, and elucidate the major components responsible for the antimalarial, antitrypanosomal, and antibacterial potencies of this plant.

## Figures and Tables

**Table 1:** Bioactive components of the methanolic extract of *Callistemon citrinus*.

Name of compounds	RI	Peak area (%)	MF	MW	Compound nature
4-Carene	919	0.22	C_10_H_16_	136	Monoterpene
Eucalyptol	1059	2.22	C_10_H_18_O	154	Oxygenated monoterpenoid
2-Nonenal	1112	0.27	C_9_H_16_O	140	Unsaturated aldehyde
Limonene diepoxide	1128	0.31	C_10_H_16_O_2_	168	Oxygenated monoterpenoid
Pinocarveol	1131	0.46	C_10_H_16_O	152	Oxygenated monoterpenoid
*α*-Terpineol	1143	1.22	C_10_H_18_O	154	Oxygenated monoterpenoid
Isopulegone, 4-methyl	1252	0.27	C_10_H_18_O	166	Oxygenated monoterpenoid
Trans-2-decenol	1266	0.92	C_10_H_20_O	156	Unsaturated alcohol
Aromadendrene	1386	0.46	C_15_H_24_	204	Sesquiterpene
(8E, 10Z)-1,8-Pentadecatriene	1518	27.00	C_15_H_26_	248	Hydrocarbon
*β*-Eudesmol	1593	0.55	C_15_H_26_O	222	Oxygenated sesquiterpenoid
Methyl-14-methyl pentadecanoate	1814	2.04	C_17_H_34_O_2_	270	Ester
Palmitic acid	1968	32.87	C_16_H_32_O_2_	256	Fatty acid
9-Octadecenal	2007	0.91	C_18_H_34_O	266	Unsaturated aldehyde
Stearic acid, methyl ester	2077	1.07	C_19_H_38_O_2_	298	Ester
Trans-vaccenic acid, methyl ester	2085	3.46	C_19_H_36_O_2_	296	Ester
Methyl linolelaidate	2093	1.49	C_19_H_34_O_2_	294	Ester
Stearic acid	2167	13.59	C_18_H_36_O_2_	284	Fatty acid
Oleic acid	2175	6.42	C_18_H_34_O_2_	282	Fatty acid
Squalene	2914	2.98	C_30_H_50_	410	Triterpene

MF = molecular formula, MW = molecular weight, RI = retention indices.

**Table 2:** Bioactive components of ethyl acetate extracts of *Callistemon citrinus*.

Name of compounds	RI	Peak area (%)	MF	MW	Compound nature
4-Carene	919	0.35	C_10_H_16_	136	Monoterpene
3,7-Octadien-2-ol, 2,6-dimethyl	1041	0.31	C_10_H_18_O	154	Oxygenated monoterpenoid
Eucalyptol	1059	4.55	C_10_H_18_O	154	Oxygenated monoterpenoid
2-Nonenal	1112	1.53	C_9_H_16_O	140	Unsaturated aldehyde
Trans-pinocarveol	1131	0.69	C_10_H_16_O	152	Oxygenated monoterpenoid
*α*-Terpineol	1143	1.56	C_10_H_18_O	154	Oxygenated monoterpenoid
Myrtanal	1126	0.51	C_10_H_16_O	152	Oxygenated monoterpenoid
Isopulegone, 4-methyl	1252	0.38	C_10_H_18_O	166	Oxygenated monoterpenoid
Aromadendrene	1386	0.46	C_15_H_24_	204	Sesquiterpene
Patchulane	1393	0.29	C_15_H_26_	206	Sesquiterpene
Viridiflorol	1530	1.17	C_15_H_26_O	222	Sesquiterpene
Methyl-2-hydroxytetradecanoate	1842	7.18	C_15_H_30_O_3_	258	Hydroxyl ester
Methyl hexadecanoate	1878	3.76	C_17_H_34_O_2_	270	Ester
Palmitic acid	1968	13.57	C_16_H_32_O_2_	256	Fatty acid
9-Octadecenal	2007	1.70	C_18_H_34_O	266	Unsaturated aldehyde
Stearic acid, methyl ester	2077	2.03	C_19_H_38_O_2_	298	Ester
Trans vaccenic acid, methyl ester	2085	5.67	C_19_H_36_O_2_	296	Ester
Methyl linolelaidate	2093	2.84	C_19_H_34_O_2_	294	Ester
Stearic acid	2167	10.43	C_18_H_36_O_2_	284	Fatty acid
Oleic acid	2175	28.23	C_18_H_34_O_2_	282	Fatty acid
Sterolic acid	2184	10.25	C_18_H_32_O_2_	280	Fatty acid
Squalene	2914	2.52	C_30_H_50_	410	Triterpene

RI = retention index, MW = molecular weight, MF = molecular formula.

**Table 3:** Qualitative phytochemical screening of ethyl acetate and methanolic extracts of *Callistemon citrinus*.

Phytochemical constituents	Test	Ethyl acetate extract	Methanol extract
Saponins	Foam test	+	+
Glycosides	Keller–Killiani test	+	−
Alkaloid	Mayer's and Wagner's tests	+	+
Steroids and triterpenoids	Salkowski's test	+	+
Phenols and tannins	Ferric chloride test	+	+
Flavonoids	Shinoda test	+	+
Fats and oils	Stain test	+	+

(+) = present, (−) = absent.

**Table 4:** Quantitative phytochemical constituents of ethyl acetate and methanol extracts of *Callistemon citrinus*.

Extracts	Overall tannin content (mg·TAE/100 g)	Overall phenolic content (mg·GAE/100 g)	Overall flavonoid content (mg·RE/100 g)	Overall flavonol content (mg·RE/100 g)	Overall antioxidant capacity (mg·AA/100 g)
Ethyl acetate	12000 ± 65.34	10964.11 ± 40.22	368.12 ± 14.48	438.38 ± 11.73	1568.73 ± 61.03
Methanol	8000 ± 28.67	22511.23 ± 105.12	688.37 ± 39.92	512.90 ± 11.00	3031.53 ± 133.07

Values are mean ± SD, *n* = 3.

**Table 5:** Minimum inhibitory concentration (MIC) values (mg/mL) for ethyl acetate, methanol extracts, and standard drug.

Bacteria	Ethyl acetate leaf extract	Methanol leaf extract	Ciprofloxacin positive control	DMSO negative control
*Aeromonas hydrophila ACC*	0.025 ± 0.00	0.15 ± 0.01	0.05 ± 0.02	0.4 mL VG
*Escherichia coli ATCC 35150*	0.100 ± 0.00	0.100 ± 0.00	0.05 ± 0.00	0.4 mL VG
*Vibro alginolyticus DSM 2171*	0.025 ± 0.00	0.100 ± 0.00	0.05 ± 0.01	0.4 mL VG
*Salmonella typhi ACC*	0.025 ± 0.01	0.025 ± 0.00	0.05 ± 0.01	0.4 mL VG
*Pseudomonas aeruginosa ACC*	0.100 ± 0.00	0.100 ± 0.00	0.05 ± 0.02	0.4 mL VG
*Staphylococcal enteritis ACC*	0.025 ± 0.01	0.100 ± 0.00	0.05 ± 0.00	0.4 mL VG
*Staphylococcus aureus ACC*	0.100 ± 0.00	0.025 ± 0.01	0.05 ± 0.01	0.4 mL VG
*Listeria monocytogenes ACC*	0.025 ± 0.00	0.100 ± 0.01	0.05 ± 0.01	0.4 mL VG

ACC = AEMREG culture collection; ATCC = American type collection center; VG = visible growth. Values are mean ± SD, *n* = 2.

**Table 6:** Minimum bactericidal concentration (MBC) values (mg/mL) for ethyl acetate, methanol extracts and standard drug.

Bacteria	Ethyl acetate leaf extract	Methanol leaf extract	Ciprofloxacin positive control	DMSO negative control
*Aeromonas hydrophila ACC*	Bacteriostatic at 0.025 ± 0.00	Bacteriostatic at 0.15 ± 0.01	Bactericidal at 0.05 ± 0.02	0.4 mL VG
*Escherichia coli ATCC 35150*	Bactericidal at 0.100 ± 0.00	Bactericidal at 0.100 ± 0.00	Bactericidal at 0.05 ± 0.00	0.4 mL VG
*Vibro alginolyticus DSM 2171*	Bacteriostatic at 0.025 ± 0.00	Bacteriostatic at 0.100 ± 0.00	Bactericidal at 0.05 ± 0.01	0.4 mL VG
*Salmonella typhi ACC*	Bacteriostatic at 0.025 ± 0.01	Bacteriostatic at 0.025 ± 0.00	Bactericidal at 0.05 ± 0.01	0.4 mL VG
*Pseudomonas aeruginosa ACC*	Bactericidal at 0.100 ± 0.00	Bactericidal at 0.100 ± 0.00	Bactericidal at 0.05 ± 0.02	0.4 mL VG
*Staphylococcal enteritis ACC*	Bacteriostatic at 0.025 ± 0.01	Bacteriostatic at 0.100 ± 0.00	Bactericidal at 0.05 ± 0.00	0.4 mL VG
*Staphylococcus aureus ACC*	Bactericidal at 0.100 ± 0.00	Bacteriostatic at 0.025 ± 0.01	Bactericidal at 0.05 ± 0.01	0.4 mL VG
*Listeria monocytogenes ACC*	Bacteriostatic at 0.025 ± 0.00	Bacteriostatic at 0.100 ± 0.01	Bactericidal at 0.05 ± 0.01	0.4 mL VG

VG = visible growth; NVG = no visible growth.

## Data Availability

The data used to support the findings of this study are available from the corresponding author upon request.

## Abbreviations

*C. citrinus*: *Callistemon citrinus*
ACC: AEMREG culture collection
ATCC: American type collection center
TFC: Total flavonoids content
TPC: Total phenolic content
TAC: Total antioxidant capacity
TTC: Total tannin content
TFlC: Total flavonol content
DPPH: 2,2-Diphenyl-1-picrylhydrazyl (DPPH)
ABTS: 2,2′-Azino-bis (3-ethylbenzthiazoline-6-sulfonic acid)
MIC: Minimum inhibitory concentration
GAE: Gallic acid equivalent
TAE: Tannin acid equivalent
QE: Quercetin equivalent.
